# Cognitive flexibility and religious disbelief

**DOI:** 10.1007/s00426-018-1034-3

**Published:** 2018-06-11

**Authors:** Leor Zmigrod, P. Jason Rentfrow, Sharon Zmigrod, Trevor W. Robbins

**Affiliations:** 1 Department of Psychology, University of Cambridge, Cambridge, CB2 3EB UK; 2 Behavioural and Clinical Neuroscience Institute, University of Cambridge, Cambridge, UK; 3 Leiden University Institute for Psychological Research, Leiden Institute for Brain and Cognition, Leiden, The Netherlands

## Abstract

Cognitive flexibility is operationalized in the neuropsychological literature as the ability to shift between modes of thinking and adapt to novel or changing environments. Religious belief systems consist of strict rules and rituals that offer adherents certainty, consistency, and stability. Consequently, we hypothesized that religious adherence and practice of repetitive religious rituals may be related to the persistence versus flexibility of one’s cognition. The present study investigated the extent to which tendencies towards cognitive flexibility versus persistence are related to three facets of religious life: religious affiliation, religious practice, and religious upbringing. In a large sample (*N* = 744), we found that religious disbelief was related to cognitive flexibility across three independent behavioural measures: the Wisconsin Card Sorting Test, Remote Associates Test, and Alternative Uses Test. Furthermore, lower frequency of religious service attendance was related to cognitive flexibility. When analysing participants’ religious upbringing in relation to their current religious affiliation, it was manifest that current affiliation was more influential than religious upbringing in all the measured facets of cognitive flexibility. The findings indicate that religious affiliation and engagement may shape and be shaped by cognitive control styles towards flexibility versus persistence, highlighting the tight links between flexibility of thought and religious ideologies.

## Introduction

The last decade has seen the birth of a new field, the ‘cognitive science of religion’ (Boyer, 2008; Whitehouse, 2004), which has illustrated that religious beliefs and traditions originate from ordinary psychological functions (Banerjee & Bloom, 2014; Heywood & Bering, 2014; Norenzayan & Gervais, 2013; Norenzayan, 2016). There is a range of human cognitive biases that are thought to make children and adults “intuitive theists” (Järnefelt, Canfield, & Kelemen, 2015; Kelemen, 2004; Kelemen & Rosset, 2009). These include human tendencies towards anthropomorphism (Epley, Waytz, & Cacioppo, 2007) and teleological thinking (Banerjee & Bloom, 2014; Heywood & Bering, 2014), which may give rise to specific religious beliefs about supernatural agents and creationism (Norenzayan, 2016).

Notably, however, research on the links between religious beliefs and cognitive processes has been largely motivated by researchers’ interest in the *content *of religious beliefs, that is, in why religions tend to depict supernatural agents or include beliefs about agentic, omniscient, and punishing gods (Norenazayan, 2016). Nevertheless, there has been little scientific attention to how the *strictness* of religious ideologies—regardless of the content of their doctrine—might also be rooted in and have consequences for cognition.

The building blocks of religious belief systems consist of strict rules and rituals that offer adherents a sense of coherence and certainty and a firm knowledge structure about the world (Atran, 2002; Dechesne et al., 2003; Epley et al., 2007; Kay, Gaucher, Napier, Callan, & Laurin, 2008; Kay, Whitson, Gaucher, & Galinsky, 2009; McCullough & Willoughby, 2009; Norenzayan & Hansen, 2006; Rutjens, Van Der Pligt, & Van Harreveld, 2010; Vail, Rothschild, Weise, Solomon, Pyszczynski, & Greenberg, 2010). Despite the proliferation in academic research into the cognitive, affective, and moral underpinnings of religiosity (Kapogiannis, Barbey, Su, Zamboni, Krueger, & Grafman, 2009; Kapogiannis, Deshpande, Krueger, Thornburg, & Grafman, 2014; Norenzayan, Shariff, & Gervais, 2016; Pennycook, Cheyne, Seli, Koehler, & Fugelsang, 2012; Purzycki et al., 2016; Rand, Dreber, Haque, Kane, Nowak, & Coakley, 2014), two important questions have not been addressed empirically: first, given the strictness of many religious ideologies, to what extent does religious adherence and practice of repetitive religious rituals shape the persistence versus flexibility of one’s cognition? Second, to what extent does early religious upbringing shape later cognitive persistence and flexibility?

When facing dynamically changing environments, human goal-directed action is thought to be characterized by a conflict between two antagonistic cognitive modes (Dreisbach & Goschke, 2004; Goschke, 2003; Hommel, 2015). On one hand, goal-directed behaviour requires that stable goals are maintained and that these are shielded from irrelevant information or competing goals. That is, it is adaptive to have an orientation towards *cognitive persistence*. On the other hand, behaviour must remain sensitive to alternative possibilities, to disengage from ineffective goals, and to flexibly adapt when environments or internal states change. Goal-directed behaviour therefore also benefits from *cognitive flexibility*. These two cognitive control modes serve antagonistic adaptive functions and have complementary advantages and disadvantages (Goschke & Bolte, 2014). Excessive shielding of goals against distraction or competing responses through cognitive persistence enhances stability, but can give rise to inflexible perseverative behaviour. In turn, excessive flexibility and behavioural switching may lead to unproductive distractibility.

Notably, individuals differ in their cognitive control tendencies towards persistence or flexibility, and there is evidence that genetic and cultural factors shape these cognitive control preferences (for review see: Hommel & Colzato, 2017). Given that religious ideologies tend to possess firm and persistent representations of how the world is structured, what is good and true, and how individuals ought to behave, it is valuable to investigate the links between religion and cognitive flexibility, as well as whether growing up with strict rules for behaviour and thought shapes cognitive persistence.

Cognitive flexibility is operationalized in the psychological and neuroscientific literature as the ability to shift between modes of thinking and adapt to novel or changing environments (Cools & Robbins, 2004; Kehagia, Murray, & Robbins, 2010). Eslinger and Grattan (1993) suggested there are at least two facets to cognitive flexibility: *reactive* flexibility, which refers to the readiness to shift behavioural responses in reaction to external cues and changing situational demands, and *spontaneous* flexibility, which refers to the ability to generate diverse and novel ideas, typically in response to a single question. Eslinger and Grattan (1993) noted that a classic measure of reactive flexibility is the Wisconsin Card Sorting Test (WCST; Grant & Berg, 1948; Heaton, 1981), which assesses individuals’ adaptation to changes in newly learnt rules and reward contingencies, and therefore the ease with which they can alternate between categories when it is no longer rewarding to persist with a previously rewarded category. Spontaneous cognitive flexibility is measured with divergent thinking tasks (Eslinger & Grattan, 1993; Tomer, Fisher, Giladi, & Aharon-Peretz, 2002), typically with the flexibility measure of the Alternative Uses Task (AUT; Guilford, 1967, 1971; Ionescu, 2012; Roberts et al., 2017). In the AUT, participants are asked to generate as many conventional and unconventional uses for familiar objects, such as a tyre or a paper clip. Reactive and spontaneous flexibility has been behaviourally and neurally dissociated in previous empirical work (e.g. Cools, Brouwer, De Jong, & Slooff, 2000; Parkin & Lawrence, 1994; Tomer et al., 2002, 2007). An additional measure of cognitive flexibility is the Remote Associates Test (RAT; Mednick, 1962, 1968), which tests the flexibility of one’s semantic networks by assessing individuals’ capacity to flexibly retrieve semantic associations between remote conceptual representations (Alexander, Hillier, Smith, Tivarus, & Beversdorf, 2007; Isen, 1990; Ishizuka, Hillier, & Beversdorf, 2007; Zmigrod & Zmigrod, 2016; Zmigrod, Zmigrod, & Hommel, 2015). The RAT may be conceptualized as merging elements of reactive and spontaneous cognitive flexibility, as it tests the way in which participants flexibly search internal conceptual networks in response to convergent external cues, and their ability to reactively restructure their thinking when they identify semantic connections between some, but not all of the cue words (Isen, 1990). The RAT is therefore a valuable complementary index of cognitive flexibility to the WCST and AUT.

Graham and Haidt (2010) drew the fruitful analogy that overemphasis on the role of belief in Gods when investigating the psychology of religions is “like focusing on the football: it seems to be where the action is, but if you stare too long at it, you miss the deeper purpose of the game, which is the strengthening of a community” (p. 140). Indeed, a concentrated focus on the content of religious beliefs can obscure key features of religious ideologies. However, in addition to studying the community and the *social* functions of religion, it is also essential to investigate the *cognitive* functions and consequences of religions. That is, if we extend the metaphor, looking at the community still misses the complete picture because one also needs to look at how playing the game shapes the minds and brains of the players, or attracts players with particular psychological characteristics.

The present study therefore sought to investigate the extent to which tendencies towards cognitive modes of flexibility versus persistence are related to three facets of religious life: (1) religious affiliation (i.e. identifying as religious or nonreligious), (2) religious practice, and (3) religious upbringing, in a sample of diverse religious ideologies.

## Methods

### Participants

The sample consisted of 744 participants (55.5% female; age: M = 36.56, SD = 13.45) recruited through Amazon Mechanical Turk and social media and were financially compensated for their participation. Participants provided their informed consent in accordance with the University of Cambridge’s Department of Psychology Ethics Committee. The majority of participants are US residents (92.2%), with 5.4% not residing in the US, and 2.4% preferring not to indicate. In terms of religiosity, 62.5% of the sample reported being religious (*N* = 465), 31.9% reported being nonreligious (atheist or agnostic, *N* = 237), and 5.6% declined to respond or did not know (*N* = 42). Out of those who reported being religious, 45.8% were Protestant Christian, 26.7% were Roman Catholic, 5.8% were Jewish, 3.7% were Hindu, 1.5% were Greek or Russian Orthodox, 1.5% were Mormon, 1.1% were Muslim, and 13.7% affiliated with other religions. In terms of frequency of religious services attendance amongst religious participants, 29.7% attended 1–2 times per week, 13.1% attended 1–2 times per month, 18.6% attended 1–2 times per year, 18.4% seldom attended, and 20.2% never attended. Across all participants, 59.4% had been raised in a home described as religious.

### Measures and procedure

#### Religiosity measures

Participants were asked the following questions, all of which were answered in a multiple-choice format with appropriate potential answers and always the option not to respond: (Q1) “What is your present religion, if any?”. Participants were presented with the following response options: “Protestant (Baptist, Methodist, Non-denominational, Lutheran, Prebysterian, Pentacostal, Episcopalian, Reformed, Church of Christ, etc.)”, “Roman Catholic”, “Mormon”, “Orthodox (Greek, Russian, or some other orthodox church)”, “Muslim”, “Jewish”, “Hindu”, “Jehova’s Witness”, “Atheist (do not believe in God)”, “Agnostic (not sure if there is a God)”, “Don’t know”, “Would rather not say”, “Other” (with option to fill in text). (Q2) “As a child, were you raised in a religious home?”. Participants could select between: “Yes”, “No”, “Don’t know”. (Q3) “Aside from weddings and funerals, how often do you attend religious services?”. Participants could select between the following responses: “More than once a week”, “Once a week”, “Once or twice a month”, “A few times a year”, “Seldom”, “Never”.

#### Wisconsin card sorting test (WCST)

The WCST (Grant & Berg, 1948) was administered with Inquisit 5 by Millisecond Software in standard fashion (Heaton, 1981, 1993). Participants are presented with four key cards and a deck of response cards that vary on three dimensions (colour, shape, and number of geometric figures) and are asked to match a fifth card from the sequentially presented response cards to one of the four key cards. Participants need to identify the correct classification rule (out of three potential rules: matching by colour, shape, or number) according to the feedback they receive after each trial. They are informed that the classification rule may change without warning, and indeed the rule alternates after participants correctly respond to ten consecutive trials, requiring a flexible set shift. The task ends after participants complete six categories (twice for each of the three rules) or after 128 trials. To index participants’ performance, the WCST accuracy rate was computed.

#### Remote associates test (RAT)

The RAT (Mednick, 1968) consisted of 15 compound remote associate problems, in which participants are presented with three cue words (e.g. cottage, swiss, and cake), and must generate the compound word solution that connects these three words (e.g. cheese). Items of varying difficulty levels were selected from established remote associate problems (Bowden & Jung-Beeman, 2003). Participants were given 20 s to respond to each item.

#### Alternative uses task (AUT)

In the AUT (Guildford, 1967), participants were asked to generate as many possible uses for two common household items (brick and newspaper) for 2 min. Participants’ responses were recorded and scored along four components by two independent raters in accordance with previous guidelines (Cronbach’s alpha = 0.994; Chermahini & Hommel, 2010; Madore, Addis, & Schacter, 2015; Roberts et al., 2017). Flexibility was scored according to the number of distinct categories that participants’ responses for a given item could be clustered into (e.g. using a newspaper for making origami and making paper dolls are uses that would fall under the same category of arts and crafts, while using a brick for swatting a fly would fall under a separate category). The total flexibility score comprised the sum from all trials. Fluency constituted the total number of appropriate responses. Elaboration reflected the amount of detail provided by the participants (for brick, “build” would receive a score of 0; “build a house” would receive a score of 1; and “a weapon to protect family when your house is robbed” would be awarded 2 points for specifying detailed use and context). To score originality, each response was compared to the responses from the rest of the participants, such that responses to a given object that were only provided by 5% of the sample received an originality point. The total originality score reflected the sum of original scores per participant across all trials. To establish inter-rater reliability for appropriate categories, level of detail for the elaboration scoring, and originality, the raters separately scored 25 random participants’ responses, and once high inter-rater reliability was established with this set on all AUT measures (Cronbach’s alpha > 0.91 on all measures); the raters independently scored the rest of the participants. Each AUT measure reflects the mean score given by the two independent raters.

#### Additional measures

Additional measures that were included in this study but are not reported here included: political affiliation and conservatism (Everett, 2013), identity fusion (Jimenez, Gomez, Buhrmester, Vázquez, Whitehouse, & Swann, 2016; Swann, Gómez, Seyle, Morales, & Huici, 2009) and support for extreme pro-group actions (Swann, Gómez, Dovidio, Hart, & Jetten, 2010). The findings associated with these measures are reported and published elsewhere.

## Results

Correlational analysis revealed significant positive correlations between the three cognitive flexibility measures: *r* = .135 (*p* = .010) between WCST and RAT, *r* = .176 (*p* < .001) between AUT Flexibility and RAT, and *r* = .289 (*p* < .001) between WCST and AUT Flexibility. Given the different demands that each of these tasks makes on participants’ working memory, perception, and linguistic skills, this corroborates past work suggesting that these three measures are related, but separable facets of flexible cognition (e.g. Eslinger & Grattan, 1993; Parkin & Lawrence, 1994).

In terms of the relationship between the cognitive flexibility measures and demographic variables, age was positively correlated with RAT performance, *r* = .138 (*p* < .001), but not with WCST, *r* = −.049 (*p* = .331), or AUT Flexibility, *r* = −.051 (*p* = .186). Educational attainment was not correlated with any of the three measures: WCST, *r* = .017 (*p* = .737); RAT, *r* = .057 (*p* = .146), and AUT Flexibility, *r* = .031 (*p* = .420). There were also no differences according to gender on the three measures: WCST, *t*(393) = −.013 (*p* = .990); RAT, *t*(639) = 1.440 (*p* = .150), and AUT Flexibility, *t*(671) = −.325 (*p* = .745).

An independent samples *t* test demonstrated there was a significant difference in the age of religious participants (M = 37.83, SD = 13.44, *N* = 447) and nonreligious participants (M = 34.07, SD = 12.89, *N* = 235); *t(*680) = −3.516, *p* < .001. A Chi-Square test demonstrated an association between gender and religious affiliation, *χ*^2^(1) = 12.538, *p* < .001, such that females tended to be more religious than males. There were no differences in educational attainment of religious and nonreligious participants, *t*(687) = −1.086, *p* = .278.

To make sure that any detected differences in cognitive flexibility according to religiosity are not due to differences in these demographic variables, the variables of age, gender, and educational attainment were included as covariates in all analyses, unless otherwise stated. Furthermore, since not all participants completed the WCST, the ANCOVAs and Bonferroni corrections are reported separately for each of the cognitive flexibility measures, so that each analysis reflects the full number of participants who completed that cognitive flexibility measure.

### Religious affiliation and flexibility

**Fig. 1:**
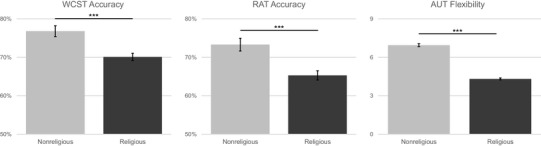
Comparison of religious and nonreligious participants on the Wisconsin Card Sorting Test (WCST), Remote Associates Test (RAT) and Alternative Uses Test (AUT) Flexibility, controlling for age, gender, and educational attainment. ***p < .001, error bars denote 1 ± standard error

Univariate ANCOVAs were computed on measures of cognitive flexibility, with age, gender, and educational attainment as covariates, and religious versus nonreligious identity as the fixed factor. An ANCOVA on WCST accuracy rate revealed a significant main effect of religious identity, *F*(1,368) = 15.425, *p* < .001, *η*_p_^2^ =0.040, such that nonreligious participants (*N* = 114) possessed higher scores on the WCST overall than religious participants (*N* = 259) (see Fig. 1). There were no significant effects of age, gender, or educational attainment. The effects of religious affiliation on WCST remain unaffected when the analysis is conducted without inclusion of the covariates: *F*(1,379) = 17.238, *p* < .001, *η*_p_^2^ =0.044.

This pattern of results was also evident in the ANCOVA on RAT accuracy rate, *F*(1,594) = 14.686, *p* < .001, *η*_p_^2^ =0.024: as seen in Fig. 1, nonreligious participants (*N* = 208) provided more correct answers on the RAT than religious participants (*N* = 391). There was a main effect of age for RAT performance, *F*(1,594) = 14.141, *p* < .001, *η*_p_^2^ = 0.023, with older participants scoring more highly on the RAT. There were no statistically significant effects of gender or educational attainment. The effects of religious affiliation on RAT performance remain unaffected when the analysis is conducted without inclusion of the covariates: *F*(1,607) = 9.066, *p* = .003.

A MANCOVA on the four AUT measures, with age, gender, and educational attainment as covariates, and religious versus nonreligious identity as the fixed factor, found a significant main effect of religious identity for the AUT Flexibility score, *F*(1,623) = 352.987, *p* < .001, *η*_p_^2^ = 0.362, and the AUT Originality score, *F*(1,623) = 69.855, *p* < .001, *η*_p_^2^ = 0.101, but not for the AUT Elaboration score, *F*(1,623) = .047, *p* = .829 or AUT Fluency score, *F*(1,623) = 2.405, *p* = .121 (Fig. 1). Specifically, nonreligious participants (*N* = 416) provided more flexible and original responses on the AUT than religious participants (*N* = 212). There was no significant effect of gender (*p* = .332) or age (*p* = .948) for the AUT Flexibility score, and a small significant effect of educational attainment, *F*(1,623) = 4.846, *p* = .028, *η*_p_^2^ = 0.008, with higher educational attainment relating to more flexible scores. For the AUT Originality score, there was no significant effect of age (*p* = .992) or educational attainment (*p* = .059), and females provided more original responses than males, *F*(1,623) = 9.222, *p* = .002. The effects of religious affiliation on AUT performance remain unchanged when the analyses are conducted without inclusion of the covariates: there is a significant effect for AUT Flexibility, *F*(1,623) = 363.404, *p* < .001, and AUT Originality, *F*(1,623) = 64.706, *p* < .001, and nonsignificant for AUT Elaboration, *F*(1,623) = .300, *p* = .584, and AUT Fluency, *F*(1,623) = .870, *p* = .351.

### Religious practice and flexibility

**Fig. 2:**
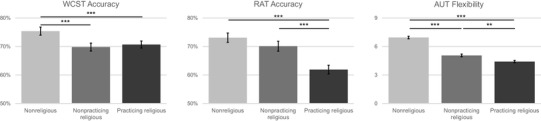
Comparison of nonreligious participants, religious participants who seldom or never attend religious services (nonpracticing), and religious participants who regularly attend religious services (practicing) on the Wisconsin Card Sorting Test (WCST), Remote Associates Test (RAT) and Alternative Uses Test (AUT) Flexibility, Bonferroni-corrected, controlling for age, gender, and educational attainment. *p < .05, **p < .01, ***p < .001, error bars denote 1 ± standard error

Participants were split into three groups according to their response to the question of frequency of religious service attendance: (1) nonreligious participants, (2) religious participants who regularly attend religious services (between multiple times per week and multiple times per year), and (3) religious participants who seldom or never attend religious services aside from weddings and funerals. Univariate ANCOVA, with age, gender, and educational attainments as covariates, showed significant differences between the three groups on the WCST, *F*(2, 384) = 7.548, *p* = .001, *η*_p_^2^ = 0.038, such that nonreligious participants (M = 76.78%, SD = 10.56%, N = 114) performed significantly better than both practicing religious participants (M = 70.77%, SD = 16.68%, *N* = 151) and non-practicing religious participants (M = 69.72%, SD = 15.12%, *N* = 125), and there were no significant differences between the two groups of religious participants (see Fig. 2), as confirmed with post hoc Bonferroni correction. There was no significant effect of age, gender, or educational attainment (*p* > .250). However, when splitting the practicing religious participants according to the frequency of their religious service attendance, significant differences emerged between those that attend religious services 1–2 per week and those that attend services 1–2 per year: participants who attend religious services weekly (M = 67.48%, SD = 17.87%) performed significantly more poorly than religious individuals who attend yearly (M = 73.95%, SD = 14.03%), *t*(117) = − 2.207, *p* = .029.

Furthermore, a univariate ANCOVA on RAT accuracy rate demonstrated a main effect of religious practice, *F*(2,631) = 13.935, *p* < .001, *η*_p_^2^ =0.042, with non-practicing religious participants (M = 72.12%, SD = 20.89%, *N* = 208) exhibiting significant greater cognitive flexibility on the RAT than practicing religious participants (M = 61.87%, SD = 27.33%, *N* = 239), and with no difference in performance between non-practicing religious participants (M = 71.23%, SD = 21.10%, *N* = 190) and nonreligious participants, as confirmed with Bonferroni correction. There was a significant effect of age, *F*(1,631) = 10.138, *p* = .002, *η*_p_^2^ = 0.016, and no significant effects of gender or educational attainment.

A MANCOVA on the four AUT measures demonstrated significant differences between the three groups in the AUT Flexibility score, *F*(2,662) = 99.688, *p* < .001, *η*_p_^2^ = 0.231, and AUT Originality score, *F*(2,662) = 26.525, *p* < .001, *η*_p_^2^ = 0.074, but not in AUT Elaboration, *F*(2,662) = .369, *p* = .692, or AUT Fluency, *F*(2,662) = 1.037, *p* = .355. Nonreligious participants exhibited higher flexibility in their AUT responses than non-practicing religious participants, which in turn provided more flexible responses than practicing religious participants (Fig. 2), after Bonferroni correction and with no significant effects of age (*p* = .519), gender (*p* = .126), or educational attainment (*p* = .098). Similarly, nonreligious participants (M = 8.72, SD = 3.94, *N* = 212) offered more original responses to the AUT than non-practicing (*p* < .001; M = 6.91, SD = 4.26, *N* = 195) and practicing (*p* < .001; M = 6.24, SD = 3.78, *N* = 261) religious participants, but Bonferroni correction revealed there were no significant differences between non-practicing and practicing religious participants in AUT Originality (*p* = .201). There was no effect of age (*p* = .822), and a significant effect of gender, *F*(1,662) = 9.357, *p* = .002, and educational attainment, *F*(1,662) = 4.394, *p* = .036, such that females and participants with higher levels of educational attainment offered more original responses.

### Religious upbringing and flexibility

Participants were split into four groups: nonreligious individuals without a religious upbringing (*N* = 109), nonreligious individuals with a religious upbringing (*N* = 101), religious individuals without a religious upbringing (*N* = 131), and religious individuals with a religious upbringing (*N* = 278). Univariate ANCOVA, with age, gender, and educational attainment as covariates, demonstrated significant differences between groups for WCST accuracy rate, *F*(3,362) = 5.207, *p* = .002, *η*_p_^2^ = 0.041, where nonreligious participants performed significantly better than religious participants regardless of upbringing after Bonferroni correction. There were no significant effects of gender (*p* = .563), age (*p* = .503), or educational attainment (*p* = .376). The same pattern of results was evident for the ANCOVA for RAT accuracy rate, *F*(3,584) = 5.248, *p* = .001, *η*_p_^2^ = 0.026, with nonreligious participants performing better than religious participants, as confirmed with Bonferroni correction. There was no effect of educational attainment (*p* = .242) or gender (*p* = .085), but there was an effect of age (*p* < .001) whereby older participants performed better on the RAT. Notably, a trend emerged in RAT performance where nonreligious participants with a religious upbringing (M = 74.50%, SD = 18.05%) performed better than nonreligious participants without a religious upbringing (M = 69.70%, SD = 23.27%), but an independent samples *t* test found that this did not achieve statistical significance [*t*(207) = − 1.647, *p* = .095].

**Fig. 3:**
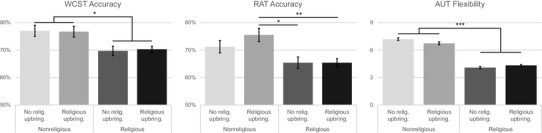
Comparison of religious and nonreligious participants with and without a religious upbringing on the Wisconsin Card Sorting Test (WCST), Remote Associates Test (RAT) and Alternative Uses Test (AUT) Flexibility, Bonferroni-corrected, controlling for age, gender, and educational attainment. *p < .05, **p < .01, ***p < .001, error bars denote 1 ± standard error. *Religious upbring*. religious upbringing, *No relig upbring*. no religious upbringing

A MANCOVA on the AUT subscores demonstrated significant differences between these four participant groups in AUT Flexibility, *F*(3,612) = 141.846, *p* < .001, *η*_p_^2^ = 0.410, and AUT Originality, *F*(3,612) = 26.236, *p* < .001, *η*_p_^2^ = 0.114, but not in AUT Elaboration, *F*(3,612) = .128, *p* = .944, or AUT Fluency, *F*(3,612) = 2.422, *p* = .065. For both the AUT Flexibility ad AUT Originality scores, nonreligious participants performed significantly better than religious participants regardless of upbringing after Bonferroni correction. As evident in Fig. 3, nonreligious participants provided significantly more flexible responses than religious participants, with no significant effect of age (*p* = .679) or age (*p* = .358), and a significant effect of educational attainment, *F*(1,612) = 7.774, *p* = .005, *η*_p_^2^ = 0.013, such that higher educational attainment was related to more flexible responses in the AUT.

## Discussion

The present study examined the relationship between three aspects of religious life: religious affiliation, practice, and upbringing, and three psychological measures of cognitive flexibility. Overall, the results suggest that religious disbelief and reduced religious practice among religious individuals are related to heightened cognitive flexibility across three independent behavioural neuropsychological measures. In terms of religious affiliation, the findings indicate that individuals who identified as nonreligious exhibited cognitive control biases towards cognitive flexibility in the WCST, RAT and AUT, while religious individuals displayed tendencies towards cognitive persistence (Fig. 1). With respect to WCST performance, this indicates that religious participants exhibited greater cognitive persistence while nonreligious participants demonstrated greater cognitive flexibility and did not persist with the previously rewarded strategy when it was no longer adaptive. In terms of RAT performance, the findings signify that nonreligious individuals tended to flexibly retrieve remote associations between conceptual representations, suggesting they possess looser boundaries between representational categories in their underlying semantic networks and a tendency to restructure thought when certain semantic searches are unproductive or misleading. The same pattern was evident in the AUT, in which nonreligious participants provided responses that spanned a more flexible range of ideas and conceptual categories for possible object uses. These findings suggest that there is a relationship between cognitive flexibility and the religious ideologies to which we adhere.

In terms of frequency of religious service attendance, there were significant differences between nonreligious individuals, religious individuals who seldom or never attend religious services (non-practicing), and religious individuals who regularly attend religious services (practicing) in terms of the AUT Flexibility score (Fig. 2). Nonreligious participants provided significantly more flexible responses than non-practicing religious participants, who in turn exhibited greater flexibility on the AUT than practicing religious participants. This linear relationship suggests that religious affiliation and religious practice may both exert an effect on the spontaneous flexibility measured by the AUT, or that individuals who display tendencies towards spontaneous flexibility may be less likely to affiliate as religious and to engage with repetitive religious rituals. Analysis of RAT performance revealed that non-practicing religious participants exhibited the same levels of cognitive flexibility as nonreligious participants, and displayed stronger tendencies towards cognitive flexibility than practicing religious participants (Fig. 2). This suggests that engagement and practice of religious rituals and routines may shape the semantic flexibility that underpins the RAT, or that individuals with greater flexibility on the RAT are more averse to engagement in religious rituals and services. It is striking that the AUT and RAT flexibility of religious participants who regularly attend religious services differs from religious participants who do not.

The amount of religious attendance was not a differentiating factor amongst religious participants in terms of WCST performance when comparing the three groups, such that non-practicing religious participants scored the same as practicing religious participants, and both groups adopted a more persistent cognitive style than nonreligious participants (Fig. 2). However, when studying the group of religious participants who reported attending religious services regularly, a significant difference emerged between participants who attend services 1–2 times per week and those who attend services 1–2 times per year. Participants with infrequent yearly attendance exhibited heightened cognitive flexibility in the WCST, while those who attended weekly behaved in a more cognitively persistent way, suggesting that high frequency of engagement with religious rituals and traditions is linked to greater cognitive persistence amongst practicing religious individuals in the WCST. This could imply that repetitive engagement with religious doctrine has a positive effect on cognitive persistence, or that individuals who are more cognitively persistent are more attracted to the regular practice of rituals that occur at religious services.

Furthermore, when analysing participants’ religious upbringing in relation to their current religious affiliation, it was manifest that current affiliation was more influential than religious upbringing in all of the measured facets of cognitive flexibility (Fig. 3). Nonetheless, RAT performance indicated a trend in which nonreligious participants who had a religious upbringing, i.e. those that choose to ‘leave’ religion in favour of atheism, were the most cognitively flexible of the four groups, including more so than nonreligious participants with no religious upbringing. While this trend did not achieve statistical significance in the current sample, it is noteworthy for future research as it could suggest that being sceptical of one’s religious doctrine and upbringing requires significant cognitive flexibility—more so than is required to remain within one’s familiar ideologies. The finding that there are significant differences in cognitive control styles between those who chose to ‘adopt’ religion and those who chose to ‘leave’ religion in the WCST, RAT, and AUT may signify that ‘adopting’ a religious ideology is a process that makes use of heightened cognitive persistence while scepticism towards religion is tied to a tendency towards cognitive flexibility. Overall, the findings indicate that the act of choosing one’s affiliation is more indicative of one’s cognitive control style than one’s upbringing.

The present findings have multiple theoretical and methodological implications for the study of the psychology of religion. First, from a methodological standpoint, this investigation suggests that it is possible to study religious life and experiences using the methodologies of cognitive psychology, and that assessing how cognitive control styles are linked to strictness of ideology is a fruitful path for psychologists of religion to take. A rich literature on the psychology of religion has demonstrated that nonreligious individuals have a stronger tendency to inhibit intuitively compelling incorrect ideas on the Cognitive Reflections Test (Gervais & Norenzayan, 2012; Pennycook et al., 2012; Shenhav, Rand, & Greene, 2012; see meta-analysis: Pennycook, Ross, Koehler, & Fugelsang, 2016; see failures to replicate: Sanchez, Sundermeier, Gray, & Calin-Jageman, 2017; Yonker, Edman, Cresswell, & Barrett, 2016), which is thought to measure an analytical cognitive style. Nonetheless, the Cognitive Reflections Test (Frederick, 2005) only relies on three items consisting of mathematically based problems and so may confound numeracy ability. It would therefore be valuable for future work to examine the interaction between an analytic and flexible cognitive style in shaping religious beliefs and identities. Interestingly, recent cross-cultural evidence suggests that there is large variability in the relation between analytic thinking and religiosity across different countries (Gervais, van Elk, Xygalatas, McKay, Aveyard, & Bulbulia, 2018), and so it will be worthwhile to investigate whether there is cross-cultural variation in the relationship between cognitive flexibility and religiosity as well.

Importantly, research has begun to focus on the perceptual underpinnings of religiosity, indicating that the hierarchical visual perception, as measured with Navon’s (1977) global–local perception task, of atheists differs from that of neo-Calvinists (Colzato, van den Wildenberg, & Hommel, 2008; Colzato et al., 2010a), Italian Roman Catholics (Colzato et al., 2010a), Orthodox Jews (Colzato et al., 2010a), and Taiwanese Zen Buddhists (Colzato, Hommel, van den Wildenberg, & Hsieh, 2010b). This suggests that religious adherence can fundamentally shape visual attention (Hommel & Colzato, 2010). Interestingly for the present study, there is a positive relationship between individual differences in the tendency to visually encode the “bigger picture” of hierarchical visual stimuli and RAT performance (Zmigrod, Zmigrod, & Hommel, 2015), suggesting that individual and group differences in perception may lend themselves to differences in cognitive control style. Consequently, engagement in religious practices appears to shape cognitive processing at multiple levels, including perception and meta-control policies such as flexibility and persistence. This is congruent with the model presented by Hommel and Colzato (2017), which proposes that the meta-control strategies of persistence and flexibility are shaped by genetic and cultural factors as well as transient situational factors.

Second, these results may be relevant for behavioural genetics studies looking at the heritability of religiousness (Beer, Arnold, & Loehlin, 1998; Bouchard, McGue, Lykken, & Tellegen, 1999; Bouchard, Segal, Tellegen, McGue, Keyes, & Krueger, 2004; Truett, Eaves, Meyer, Heath, & Martin, 1992). Individual differences in cognitive flexibility, and specifically the WCST, RAT, and AUT Flexibility, have been linked to dopaminergic systems (Barnes, Dean, Nandam, O’Connell, & Bellgrove, 2011; Braver, Cole, & Yarkoni, 2010; Butler, McNamara, & Durso, 2007, Chermahini & Hommel, 2010; Mayseless, Uzefovsky, Shalev, Ebstein, & Shamay-Tsoory, 2013), and so perhaps future behavioural genetic and epigenetic investigations on the heritability of religiosity should investigate the role of genes implicated in dopamine functioning. In fact, an integrative predictive processing framework for understanding religion has been recently proposed (van Elk & Aleman, 2017), implicating the dopaminergic system in the maintenance of religious and paranormal beliefs (Butler, McNamara, & Durso, 2010, 2011; Krummenacher, Mohr, Haker, & Brugger, 2010; Sasaki, Kim, Mojaverian, Kelley, Park, & Janušonis, 2011; Schjødt, Stødkilde-Jørgensen, Geertz, & Roepstorff, 2008). Generating a neurobiologically-informed research agenda may therefore sharpen our understanding of how ideological commitment is biologically—and not just socially—transmitted across generations through cognitive control styles.

This investigation looked at three aspects of religious life, and this was not meant to be an exhaustive list of all the facets of religious ideologies and experiences. Consequently, future research will need to elaborate on more features of religious rituals and practice, and examine a wider range of religions than those present in this sample. It will also be valuable to examine the trade-off between cognitive flexibility and persistence to a greater extent to identify how these cognitive control modes interact (for an in-depth review, see Hommel & Colzato, 2017). Furthermore, this raises interesting questions: does a ritualistic lifestyle and adopting a firm ideological doctrine shape one’s cognitive persistence, or do individuals with heightened cognitive persistence tend to engage more with religious life? Or perhaps it is an interaction of these factors, and if so, it is valuable to characterise the interaction between cognitive predispositions and environmental influences. Longitudinal data may be the best way to address these questions.

To what extent are these results specific to religious ideologies or general to other ideological systems which are characterized by strictness of thought? Recent research suggests that individuals with strongly nationalistic attitudes also tend to exhibit lower cognitive flexibility on the WCST and RAT relative to individuals with a more fluid understanding of nationalistic identity (Zmigrod, Rentfrow, & Robbins, 2018). Furthermore, lower cognitive flexibility is related to a greater endorsement of extreme pro-group actions (such as violence against an outgroup member) to protect the nationalistic ingroup (Zmigrod, Rentfrow, & Robbins, under review). Moreover, individuals who are strongly affiliated to a political party—regardless of whether this is a traditionally conservative or liberal political party—display lower cognitive flexibility in comparison to politically moderate individuals (Zmigrod, Rentfrow, & Robbins, under review). These findings suggest that cognitive flexibility may be related to a reduced tendency to engage in ideological thinking across domains, including religion, nationalism, and politics. This may provide some hints about the directionality of these effects. Simply engaging frequently with religious rituals may not fully account for the greater cognitive persistence evident in religious individuals, since ideological systems (such as nationalism) that require less frequent and more passive engagement are also associated with cognitive persistence.

In the *Varieties of Religious Experience*, James argued that “to the psychologist, the religious propensities of man must be at least as interesting as any other of the facts pertaining to his mental constitution” (1902; p. 2). Here we find that individuals’ religious propensities may in fact be linked to features of their cognitive constitution. The results indicate that affiliation and engagement with religion may be rooted in and have consequences for cognitive control styles. These findings highlight that ideological identity, engagement, and environmental upbringing all interact to shape—and be shaped by—the characteristics of one’s cognition. This underlines the tight parallels between one’s flexibility of thought and adherence to ideologies.
